# High-resolution gene expression data from blastoderm embryos of the scuttle fly *Megaselia abdita*

**DOI:** 10.1038/sdata.2015.5

**Published:** 2015-03-03

**Authors:** Karl R Wotton, Eva Jiménez-Guri, Anton Crombach, Damjan Cicin-Sain, Johannes Jaeger

**Affiliations:** 1 EMBL/CRG Research Unit in Systems Biology, Centre for Genomic Regulation (CRG), 08003 Barcelona, Spain; 2 Universitat Pompeu Fabra (UPF), 08002 Barcelona, Spain

**Keywords:** Evolutionary developmental biology, Developmental biology, Systems biology, Gene expression analysis

## Abstract

Gap genes are involved in segment determination during early development in dipteran insects (flies, midges, and mosquitoes). We carried out a systematic quantitative comparative analysis of the gap gene network across different dipteran species. Our work provides mechanistic insights into the evolution of this pattern-forming network. As a central component of our project, we created a high-resolution quantitative spatio-temporal data set of gap and maternal co-ordinate gene expression in the blastoderm embryo of the non-drosophilid scuttle fly, *Megaselia abdita.* Our data include expression patterns in both wild-type and RNAi-treated embryos. The data—covering 10 genes, 10 time points, and over 1,000 individual embryos—consist of original embryo images, quantified expression profiles, extracted positions of expression boundaries, and integrated expression patterns, plus metadata and intermediate processing steps. These data provide a valuable resource for researchers interested in the comparative study of gene regulatory networks and pattern formation, an essential step towards a more quantitative and mechanistic understanding of developmental evolution.

## Background & Summary

Understanding the function and evolution of complex regulatory networks is a challenge that lies at the heart of modern biology^1–5^. In the context of developmental evolution (evo-devo), tackling this challenge requires systematic and quantitative comparative studies of pattern-forming gene regulatory networks. We use a combination of genetics, dynamical systems modelling and reverse engineering to achieve this aim^6,7^. As a case study, we focus on the gap gene network involved in pattern formation and segment determination during early development of the vinegar fly *Drosophila melanogaster* and other dipteran insects (flies, midges, and mosquitoes)^8^. Our approach relies on quantitative gap gene expression data with high spatial and temporal resolution, not only in standard laboratory models, but also in non-model organisms^9–19^. Such data must be based on quantitative microscopy and image bioinformatics^20–22^ to preserve the spatial aspect of gene expression, which is crucial for the study of pattern formation.

Here, we present a quantitative data set of segmentation gene expression from a non-drosophilid species, the scuttle fly *Megaselia abdita* ^23^. It comprises a high-resolution spatio-temporal atlas for the expression of the maternal-coordinate genes *bicoid (bcd), hunchback (hb),* and *caudal (cad),* as well as the gap genes *hb*, *Krüppel* (*Kr*), *knirps (kni)*, *giant* (*gt*), *tailless (tll),* and *huckebein (hkb)* along the major embryonic axis—the antero-posterior or A–P axis—during the cleavage and blastoderm stages of early development^24–26^. We are using these data, in combination with our previously published expression data set from *D. melanogaster* ^14^, to study the evolution of maternal gradients and gap gene regulation across dipteran species^27,28^.

Quantified spatio-temporal expression data form the foundation of our investigation in two complementary ways. First, high-resolution measurements of gene expression dynamics—in combination with gene knock-down by RNA interference (RNAi)—allows us to study maternal regulatory inputs^28^ and gap-gap cross-regulatory interactions^27^ at an unprecedented level of rigour and detail. Our work reveals how small changes in regulatory interactions lead to different mechanisms establishing embryo polarity and the dynamic positioning of shifting gap domain boundaries in *M. abdita* compared to *D. melanogaster*. Careful characterisation of expression timing, in combination with the dosage-dependent nature of RNAi, allows us to assess the effects of subtle changes in regulatory interaction strength on expression dynamics. This would not be possible without a high-resolution quantitative data set such as the one presented in this paper. Second, in addition to direct analysis, we also used our data to fit quantitative dynamic models of the gap gene network (publication currently under review). These models, which have been verified by RNAi knock-down, go beyond genetic analysis in that they predict specific and precise regulatory mechanisms for gap gene expression dynamics and its evolution by quantitative system drift.

**Figure 1:**
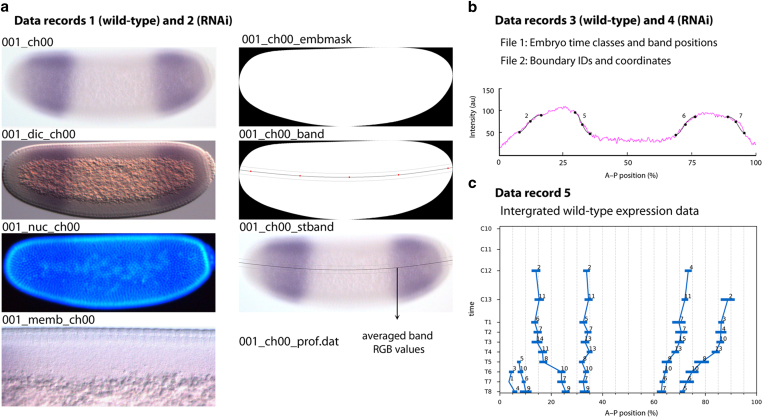
Overview of data records presented in this paper. (**a**) Data Records 1 (wild-type) and 2 (RNAi). These data records include cropped and rotated images of gap gene expression taken using bright field and differential interference contrast optics (ch00 and dic_ch00 suffixes respectively), an image of the nuclear counterstain (nuc_ch00) and an unprocessed image of the dorsal membrane morphology (memb_ch00). These data records also include embryo masks (embmask) and images of the band location superimposed onto the embryo mask (band) and brightfield (stband) images. The file 001_ch00_prof.dat contains averaged band RGB values seen plotted in magenta in (**b**). (**b**) Data Records 3 (wild-type) and 4 (RNAi): these records are composed of two CSV files that document the time class and position of the 10% strip used for profile extraction for each embryo (File 1), plus the associated boundary IDs and coordinates (File 2). The graph shows an example, where splines fit to boundaries 2, 5, 6 and 7 of *gt* are plotted against the extracted profile (magenta; see Fig. 2 for boundary numbers). Images in (**a**) and (**b**) correspond to embryo 001 that can be found in Data Record 1 in ish/megaselia/ma_gt_260911/proc. (**c**) Data Record 5. This data record is composed of a CSV file containing averaged coordinates and standard deviations of expression boundaries for each gene. Embryos are oriented with anterior to the left, dorsal up.

The expression data presented here consist of (1) images of carefully curated^21^ and staged^26^ laterally aligned *M. abdita* blastoderm embryos stained against one or two maternal co-ordinate or gap mRNA gene products by whole-mount *in situ* hybridisation^14^ in a wild-type (Fig. 1a; Data Record 1, Data Citation 1) and RNAi-treated background (Fig. 1a; Data Record 2, Data Citation 1); (2) the associated quantified spatial gene expression profiles^21^ classified by gene and developmental time point (cleavage cycles 1–14A, C1–C14A; cleavage cycle C14A is subdivided into eight time classes, C14-T1–T8)^26^ for wild-type (Fig. 1b; Data Record 3, Data Citation 1) and RNAi-treated embryos (Fig. 1b; Data Record 4, Data Citation 1); and (3) integrated spatio-temporal expression data, averaged across all embryos stained for a given gene at a given time point in wild-type embryos (Fig. 1c; Data Record 5, Data Citation 1)^27,28^.

## Methods

Blastoderm-stage embryos of *M. abdita*—corresponding to embryonic stages 4 and 5 (ref. 26)—were collected 4 h (hrs) after egg laying (see refs 29,30, for fly culture and embryo collection/fixation). Gap gene mRNA expression patterns were visualised using an enzymatic (colorimetric) *in situ* hybridisation protocol as previously described^14^. Visualisation was performed using NBT/BCIP (purple) and/or FastRed (red) staining reagents. We used single RNA probes for the majority of stains (mostly NBT/BCIP). Embryos double-stained for gap genes, or a gap gene plus the pair-rule gene *even-skipped (eve)*, were used to confirm and register the position of expression domains relative to each other.

RNAi treatment was carried out as described in refs 23,31,32. Two distinct dsRNA constructs were injected per gene, one being the knock-down agent, the other one—with conserved domains excluded—serving as a control. RNAi-treated embryos were then stained for each of the trunk gap genes as described above.

Stained wild-type and RNAi-treated embryos were imaged, staged and processed as previously described^21,27^ (Data Records 1–4, Data Citation 1). In brief, four images were taken of each stained embryo (Fig. 1a)^21^: a bright-field image from which the expression profile was extracted, a differential interference contrast (DIC) image used to create the whole-embryo mask, and two images used to determine the time class of each embryo^26^: one of fluorescently counterstained nuclei, and the other showing details of membrane morphology with DIC optics. Images of wild-type embryos were further processed through a quantification pipeline as previously described^21^. In summary, we automatically create a whole-embryo mask used for image cropping, rotation, and alignment; we then locate a 10%-strip along the dorso-ventral midline of the embryo with a cubic spline (see ref. 33 for mathematical details) that is defined by five equidistant knots—that is, five control points; knot position is automatically pre-set using a skeleton algorithm and can be manually adjusted if necessary (Fig. 1a); we extract expression profiles along the antero-posterior axis along this strip as follows (Fig. 1b): for each pixel on the spline curve we extract average RGB values, calculated across a perpendicular column spanning the entire width of the 10%-strip; intensity profiles of NBT/BCIP (purple) stains were extracted using the inverse red RGB channel (that is: intensity=255—red; the blue RGB channel was ignored as its signal is very similar to the red channel with a weaker signal-to-background ratio); the profile of FastRed (red) stains was determined by subtracting the green from the red RGB channel and inverting the outcome. In this manner both the blue component of NBT/BCIP and the yellow-like background of the unstained embryo regions are removed; once profiles are extracted, we visually identify expression domain boundaries, which are manually fitted by clamped splines (first derivative set to 0.0 at the ends of the spline) to measure their width and position (Fig. 1b). Subsequently, we calculate median boundary positions and expression variability for each boundary at each time class, which results in an integrated high-resolution data set of spatio-temporal gene expression (Fig. 1c)^27,28^. RNAi-treated embryos were processed through the same pipeline as wild-type embryos to measure the position of gene expression boundaries. Due to the variability in gene knock-down effect, we did not evaluate median boundary positions in this data set^27^.

## Data Records

### Data record 1—image data and extracted profiles from wild-type *M. abdita* embryos stained for maternal co-ordinate and gap gene expression

This data record contains series of images stored in Portable Network Graphics (PNG) format displaying wild-type *M. abdita* embryos that have been stained for gap gene expression at various intermediate stages of data processing. Each series of images comes with a data file containing an extracted expression profile. Data are archived as a ZIP-compressed file, which is organised into a hierarchy of directories (Data Record 1, Data Citation 1). The top directory ‘ish’ (*in situ* hybridisation) contains the sub-folder ‘megaselia’, which in turn contains a number of directories corresponding to individual batches of stained embryos, which are each given a specific batch ID. Each embryo within these batches is given an individual embryo ID and each processing step receives an identifying suffix in our file-naming scheme. Details are as follows.

#### Batch ID

Each batch directory is named in the following format ‘species_stain_date’. For example, ma_gt_260911 contains data from *M. abdita* (ma) stained for the gap gene *giant* (gt) on the 26th September 2011 (260911). Each of these directories contains a folder called ‘proc’ (short for stored PROCessing steps), which contains the processed images.

#### Embryo ID

Images from specific embryos within the ‘proc’ directories receive a numerical ID: 001, 002, 003... followed by a suffix that identifies each of the processing steps. Suffixes are:

#### Suffix: _ch00

Cropped and rotated bright-field images (taken with a 10X objective) used to extract gene expression boundary positions.

#### Suffix: _dic_ch00

Cropped and rotated differential interference contrast (DIC) images (taken with a 10X objective) used to create the embryo mask.

#### Suffix: _nuc_ch00

Fluorescent images (taken with a 10X objective) with DAPI nuclear counterstain used for staging embryo into separate cleavage cycles (C1–C14A).

#### Suffix: _memb_ch00

DIC images (taken with a 40X objective) of mid-dorsal membrane morphology used to classify embryos within cleavage cycle 14A (C14A) into time-classes T1–8.

#### Suffix: _embmsk

Binary whole-embryo masks generated from DIC images used to automatically crop/rotate embryo images.

#### Suffix: _band

These images show the 10%-strip (band) used to extract gene expression profiles in overlay with the binary embryo mask.

#### Suffix: _stband

These images show the 10%-strip (band) used to extract gene expression profiles in overlay with the original bright-field image from which expression profiles are extracted.

#### Suffix: _prof.dat

Text file containing extracted expression profiles along the 10%-strip. Channel extraction is performed as described above.

### Data record 2—image data and extracted profiles from RNAi-treated *M. abdita* embryos stained for maternal co-ordinate and gap gene expression

This data record contains series of images stored in Portable Network Graphics (PNG) format displaying RNAi-treated *M. abdita* embryos that have been stained for gap gene expression at various intermediate stages of data processing. Each series of images comes with a data file containing an extracted expression profile. Data are archived as a ZIP-compressed file, which is organised into a hierarchy of directories (Data Record 2, Data Citation 1). The top directory ‘RNAi’ (RNA interference) contains the sub-folder ‘megaselia’, which in turn contains a number of directories corresponding to separate RNAi experiments, which are each given a specific batch ID. Each embryo within these batches is given an individual embryo ID and each processing step receives an identifying suffix in our file naming scheme as described for data record 1.

### Data record 3—data tables for gap gene expression in wild-type embryos

These tables contain positions of extracted boundaries and associated meta-data on time classification and embryo regions used for boundary extraction. The data have been deposited with figshare as two tables in comma-separated value (CSV) format (Data Record 3, Data Citation 1).

#### File 1: wt_timeclasses_bands.csv

This file contains information on time classification and positions of 10%-strips from which expression data have been extracted from wild-type embryos. Columns are structured as follows:

1. embryo

Shows the embryo ID and its directory path.

2. timeclass

This column contains the cleavage cycle (C1 to C13), or time class if in C14A (C14_T1–T8). Our staging scheme is based on ref. 26: all cleavage cycles up to C9 are of a very similar duration (approximately 10 min). Blastoderm stages C10 to C14A last for 13, 11, 14, 23, and 58 min respectively. C14A is further subdivided into eight equal time classes (T1–8) of around 7 min each.

3. spline_height

Indicates the absolute height of the 10%-strip in pixels calculated as 10% of the height of each particular embryo mask.

4. – 13. spline_x1 to x5 and spline_y1 to y5

These columns indicate the *x* and *y* co-ordinates of the five equidistant points placed on the midline spline. Positions are given in pixels with an origin at the upper left corner of the image.

#### File 2: wt_boundary_data.csv

This file contains the co-ordinates of expression boundaries extracted by fitting clamped splines to profiles from wild-type embryos. Columns are structured as follows:

1. embryo

Shows the embryo ID and its directory path.

2. boundary_id

**Figure 2:**
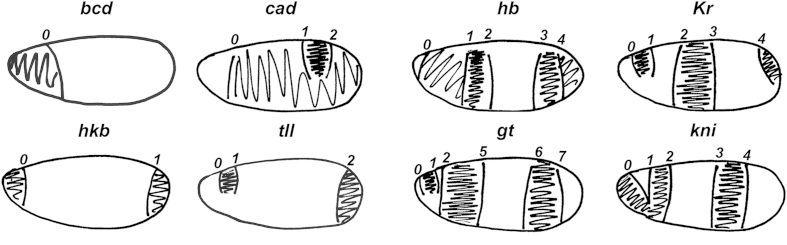
*M. abdita* boundary naming scheme. This figure displays schematic drawings of embryos showing expression boundary numbers and relative positions for each *M. abdita* gene listed in File 2 of Data Records 3 and 4. *gt* boundaries 3 and 4 are omitted to provide consistent numbering with homologous *D. melanogaster* boundaries^14^. Maternal inputs (*bcd, cad*) and terminal gap genes (*tll, hkb*) are shown on the left. Trunk gap genes (*hb, Kr, gt, kni*) are shown on the right. Embryos drawn anterior to the left, dorsal up. Figure reproduced from (ref. 27, Supplementary File 2).

This number represents a specific expression boundary as shown in Fig. 2.

3. facing

This column indicates the position of a boundary with regard to its expression domain (i.e. which direction it is facing). There are two possible values: ‘anterior’ and ‘posterior’.

4. gene

This column indicates the gene name (given as standard gene abbreviation; for example, ‘gt’ for *giant*).

5. channel

This column records the colour of the stain (purple: NBT/BCIP, or red: Fast Red) from which the boundaries were extracted.

6. – 11. boundary_x1 to x3 and boundary_y1 to y3

These columns contain the start-, mid-, and end-points of the clamped splines used to measure boundary positions and width. Positions are indicated in pixels with an origin in the upper left corner of the graph.

### Data record 4—data tables for gap gene expression in RNAi-treated embryos

These tables contain positions of extracted boundaries and associated meta-data on time classification and embryo regions used for boundary extraction. The data have been deposited with figshare as two tables in CSV format (Data Record 4, Data Citation 1).

#### File 1: RNAi_timeclasses_bands.csv

This file contains information on time classification and positions of 10%-strips from which expression data have been extracted from RNAi-treated embryos. Columns are structured as described for Data Record 3, File 1.

#### File 2: RNAi_boundary_data.csv

This file contains the co-ordinates of expression boundaries extracted by fitting clamped splines to profiles from RNAi-treated embryos. Columns are structured as described for Data Record 3, File 2.

### Data record 5—integrated wild-type expression data

These data have been deposited with figshare as a table in CSV format (Data Record 5, Data Citation 1).

#### File 1: wt_integrated_boundaries.csv

This file details averaged coordinates of expression boundaries. Averages and deviations are computed as both arithmetic means and medians, and standard deviations and median absolute deviations (MAD), respectively. The file consists of the following columns:

1. gene

This column indicates the gene name (given as standard gene abbreviation; for example, ‘gt’ for *giant*).

2. timeclass

This column contains the cleavage cycle (C1 to C13), or time class if in C14A (C14_T1–T8). Our staging scheme is based on ref. 26. See Data Record 3 for details.

3. boundary id

This number represents a specific expression boundary as shown in Fig. 2 (ref. 27).

4. mean

Contains the (arithmetic) mean A–P position (in % embryo length) of the boundary at a given time point.

5. median

Contains the median A–P position (in % embryo length) of the boundary at a given time point.

6. standard deviation

Shows the standard deviation (in % embryo length) for each boundary and time class.

7. MAD

Shows the median absolute deviation (MAD, in % embryo length) for each boundary and time class.

## Technical Validation

Our data processing pipeline^21^ produces high-quality spatio-temporal mRNA expression data, which are quality-checked and validated in several ways. Quality control is implemented as follows: embryo morphology, orientation, and quality of the staining (for example, signal-to-background ratio) are carefully assessed and recorded during data processing, and only high-quality data are selected for further use. Moreover, processing steps that are sensitive to observer bias—such as fitting of splines to extract boundaries (Fig. 1b) or time classification of embryos^26^—are always checked by two independent researchers.

In terms of data validation, a *D. melanogaster* expression data set—quantified in a manner equivalent to our *M. abdita* data—is in close agreement with previously published, independent qualitative and quantitative evidence. This includes the vast literature with qualitative observations on maternal co-ordinate and gap gene expression patterns in *D. melanogaster* that has accumulated over the past 30 years (reviewed in ref. 8) and, more importantly, a number of more recent quantitative studies documenting gap gene expression patterns in *D. melanogaster* and related drosophilid species both at the mRNA and protein level^11,12,14,17,34–36^. Another line of evidence also confirms the accuracy and validity of our data: we have used our *D. melanogaster* mRNA data set to fit gene network models of the gap gene system. The regulatory network structure predicted by these models is in strong agreement, not only with previous models fitted to protein expression data^9,10,13,14^, but also with the evidence on regulatory interactions gained from a vast amount of genetic experiments^8^. Especially the latter case, where gap gene network structures derived from completely independent and complementary evidence—gained by reverse-engineering and genetics, respectively—agree, is a strong indication that our data accurately represent the actual expression dynamics in the embryo.

Gene knock-down by RNAi is an established protocol in *M. abdita* ^23,31,32^. We control for possible off-target effect by the injection of two dsRNA fragments, one of which lacks conserved regions (that is, zinc finger or homeobox domains). In each case this resulted in identical knock-down phenotypes. Using this RNAi protocol, we have been able to reproduce previously published gene expression patterns and cuticle phenotypes in RNAi-treated *M. abdita* embryos^27,31,32^. In addition, knock-down of gap genes that had not been examined previously, closely resemble the equivalent null mutant phenotypes in *D. melanogaster* ^27^. Finally, RNAi using an identical protocol in *D. melanogaster* produced exact phenocopies of the respective null mutants^28^.

## Usage Notes

Our data set is available online through the SuperFly database: http://superfly.crg.eu (ref. 37). SuperFly provides a user-friendly web interface for browsing, searching, and downloading batches of data or individual embryos. It includes an online help system with instructions on how to use it.

## Additional information

**How to cite this article:** Wotton, K. R. *et al.* High-resolution gene expression data from blastoderm embryos of the scuttle fly *Megaselia abdita*. *Sci. Data* 2:150005 doi: 10.1038/sdata.2015.5 (2015).

## Supplementary Material
